# Research on a Three-Way Decision-Making Approach, Based on Non-Additive Measurement and Prospect Theory, and Its Application in Aviation Equipment Risk Analysis

**DOI:** 10.3390/e26070598

**Published:** 2024-07-14

**Authors:** Ruicong Xia, Sirong Tong, Qiang Wang, Bingzhen Sun, Ziling Xu, Qiuhan Liu, Jiayang Yu, Fan Wu

**Affiliations:** 1 Equipment Management and Unmanned Aerial Vehicle Engineering School, Air Force Engineering University, Xi’an 710051, China; rayc0-xia@foxmail.com (R.X.); xuziling0510@foxmail.com (Z.X.); ayden.liu@foxmail.com (Q.L.); y1312887589@163.com (J.Y.); a_17792030951@163.com (F.W.); 2 School of Economics and Management, Xidian University, Xi’an 710071, China; bzsun@xidian.edu.cn

**Keywords:** incomplete information, three-way decision-making, non-additive measure, prospect theory, aviation equipment risk analysis

## Abstract

Due to the information non-independence of attributes, combined with a complex and changeable environment, the analysis of risks faces great difficulties. In view of this problem, this paper proposes a new three-way decision-making (3WD) method, combined with prospect theory and a non-additive measure, to cope with multi-source and incomplete risk information systems. Prospect theory improves the loss function of the original 3WD model, and the combination of non-additive measurement and probability measurement provides a new perspective to understand the meaning of decision-making, which could measure the relative degree by considering expert knowledge and objective data. The theoretical basis and framework of this model are illustrated, and this model is applied to a real in-service aviation equipment structures risk evaluation problem involving multiple incomplete risk information sources. When the simulation analysis is carried out, the results show that the availability of this method is verified. This method can also evaluate and rank key risk factors in equipment structures, which provides a reliable basis for decisions in aviation safety management.

## 1. Introduction

Safety is an eternal theme in the aviation industry. As aviation technology advances and equipment designs become more complex, the demands for ensuring structural safety have also escalated. The structural safety of aviation equipment when in service is a significant issue for safeguarding human lives and property intactness, for severe structural damage always leads to catastrophic tragedy. The in-service structural safety risk evaluation of aviation equipment requires a systematic and comprehensive evaluation of the potential structural safety risks that such aviation equipment may encounter during its operational phase. The risk evaluation process mainly involves the identification and quantification of potential structural safety hazards to ensure the structural integrity and reliability of aviation equipment, which provides safety managerial personnel with a decision basis, thereby safeguarding the safety of the aircraft and its crew members.

However, the changing service environment and dynamic mission completion have brought challenges regarding the reliable risk evaluation of in-service aviation equipment structures. Moreover, due to the limitations of individual knowledge backgrounds and environments, the safety information obtained presents various characteristics, including ambiguity, incompleteness, and multi-source heterogeneity, leading to hampered accuracy and a lack of appropriate risk identification and quantification. In particular, multiple safety information sources, such as inherited risk factors from the design phase, historical unsafe event records, existing real-time operation data, etc., give rise to inevitable data heterogeneity and incompleteness. Therefore, how to evaluate risk factors comprehensively and credibly when facing uncertain circumstances is a meaningful topic of study. 

Risk evaluation as an engineering-oriented research field has developed greatly in the past 30–40 years [1,2,3,4,5]. During this period, many methods of risk assessment and risk management have been applied in different fields, including economics, new nanomaterials, engineering management, risk tolerance estimation, microbial risks, etc. Risk evaluation and management have two main types: the first is problem-driven, using risk evaluation and risk management to deal with activities in specific situations [6,7,8]; the second is model-driven, studying the concepts, frameworks, and theories of risk evaluation, and establishing risk evaluation models through such studies [3,9,10,11,12]. Li and Bi et al. [13] combined linguistic Pythagorean fuzzy sets to represent uncertain information and analyzed the maintenance risk factors of offshore wind turbines, based on their state. Commonly used methods or models include FMEA [14,15] and fault trees [16]. Yu and Yang et al. [14] used interval intuitionistic fuzzy rough numbers to represent fuzzy information. Combining ExpTODIM with PROMETHEE methods, the FMEA method was improved and then applied to the risk analysis problem of submarine oil pipelines. By combining appropriate preference information aggregation methods and evaluation methods [17,18,19,20] to select the appropriate methods and models, risk factors can be evaluated and ranked to determine the key risk factors. 

Furthermore, as a new mathematical tool for studying uncertain problems, the 3WD theory has received attention from scholars in various fields in recent years [21,22,23,24]. The theory has advantages when describing and dealing with uncertainty problems. In the face of incomplete and multi-source heterogeneous data types, the 3WD theory establishes corresponding loss functions for different objects and their three possible decisions. Based on the loss function, the expected loss situation of each decision object can be obtained. Two fields of interest in the three-way decision theory are theoretical research [21,22,23,25,26,27,28,29] and innovative applications, such as clinical diagnosis [24,27], supply chain selection [26], equipment project selection [28], etc. Meanwhile, many scholars have also adapted this theory to risk analysis. Jia et al. [30] analyzed the risk problems of urban energy networks and used a knowledge graph to identify risk factors. Du and Liu et al. [25] described risk information using multi-granularity-imbalanced language combined with three-way decision models to improve the FMEA method and proposed a new three-way FMEA model. In the existing literature, most of the researchers only concentrate on a single risk information source, seldom paying attention to multiple sources. Thus, we considered using multiple risk information sources to identify and quantify key risk factors.

In recent years, most of the research on risk decision-making has used unilateral information [31,32,33], such as using the single method to improve the FMEA method [15] or only using predictive analysis methods to predict risks. Few scholars have considered using different sources of information to deal with aviation equipment risk problems. As the complexity and quantity of data gradually increase, it is difficult to analyze them comprehensively by previous and single-analysis methods. Therefore, using data-driven methods to analyze, identify, and manage risk factors is an effective way to handle large amounts of complex data. Many scholars have conducted research into and discussions on this approach. Murugan and Sree Kala T [34] applied machine learning methods to the field of risk analysis and management. Liu and Xu et al. [32] considered the interaction between different risk factors and combined Choquet integral and fuzzy measurement techniques to improve the FMEA method, thereby proposing a new risk analysis method. Seungwon et al. [16] applied dynamic fault trees to the risk analysis problem of aircraft collisions. It is insufficient to adopt only the FMEA approach to evaluate risk; it is necessary to combine practical states and the FMEA approach when comprehensively analyzing risk. Therefore, leveraging multi-risk information sources, including FMEA results, real-time operation data, and historical safety records to evaluate in-service risks could be a more precise and reliable technique. 

To summarize, there are few studies in the aviation field about how to carry out risk evaluations when encountering multiple risk information sources. Thus, we applied a new 3WD model combining prospect theory and non-additive measurement to solve the risk evaluation problem of in-service aviation equipment’s structural safety. Considering the multiple risk information sources, including inherited risk factors from the design phase, historical unsafe event records, and existing real-time operation data, a multi-source incomplete risk information system (MSIRIS) was established. First, we defined the distance measurement on a single attribute and similarity measurements between attributes. The value of all attributes was converted to be positively correlated with risk, and risk loss was taken as the distance between attribute value and risk expectation. Minimum attribute values were used to fill attribute blanks, and attribute weight was determined by calculating information entropy to lessen the adverse influence of incomplete information. Then, risk relative utility was calculated by applying prospect theory to risk loss. Based on this utility, relative loss functions and similarity classes were determined. Three state sets were calculated using a non-additive measure, considering expert knowledge. Based on setting the decision rules, the risk factor ranking was determined, and simulation analysis with the relevant parameters was conducted. 

This paper is structured in the following sections. Section 2 introduces the mathematical theoretical basis required for this article. Section 3 describes the 3WD method, which is based on non-additive measurement and prospect theory. Section 4 introduces decision-making problems in the context of FMEA and shows the application of the method in FMEA. Section 5 introduces application examples and performs a simulation analysis based on the results. Section 6 offers our conclusions.

## 2. Preliminaries

In this section, we provide an overview of rough sets, three-way decision-making, fuzzy measurement, and Choquet integrals, which lays a mathematical theoretical foundation for the new model that is proposed below.

### 2.1. Rough Set

In this section, we will restate the rough set theory and the three-way decision-making model,

**Definition** **1** [35]**.** *Let* U  *be an infinite universe.* R *is the relation of equivalence in the universe* U*. For an arbitrary set where* X ∈U*, the lower approximation and the upper approximation with regard to* X *are represented by* $\underline{R}\left(X\right)$ *and* $\bar{R}\left(X\right)$*, which are defined as follows:*

(1) $$\underline{R}\left(X\right)=\left\{x|{\left[x\right]}_{R}\subseteq X\right\}\;\bar{R}\left(X\right)=\left\{x|{\left[x\right]}_{R}\cap X\neq \emptyset \right\}$$

We define $\left(\underline{R}\left(X\right),\bar{R}\left(X\right)\right)$ as a rough set, based on the binary relation R in set X.

### 2.2. Three-Way Decision-Making

Three-way decision-making is an extension of traditional binary decision processes, where decisions are not only limited to acceptance or rejection but also include a non-commitment option: 

Acceptance: This decision is made when there is sufficient evidence or confidence that the outcome is favorable or as expected. It is a commitment to a particular course of action.

Rejection: This decision is taken when the evidence or confidence is low, indicating that the outcome is not favorable or as expected. It is a decision to avoid a particular course of action.

Non-Commitment: This is a decision to neither accept nor reject a course of action due to insufficient evidence or high uncertainty. It allows for further investigation, gathering more information, or waiting for conditions to become clearer before making a definitive decision.

This approach is particularly useful in situations involving high levels of uncertainty, as it allows for a more nuanced and cautious decision-making process. It has been applied in various fields, including reliability management, expert systems, medical diagnosis, etc. [21,23,24,25,31].

### 2.3. Fuzzy Measurement

The classical theory of measurement is mainly based on additive measures, but in recent years, when faced with a complex and changeable decision-making environment, the classical additive measures are far from satisfactory, which leads to errors in the conditions of the measurements. Based on this point of view, this paper adopts the set function of Sugeno non-additive measurements, i.e., fuzzy measurements, and restates the definition of fuzzy measurements.

**Definition** **2** [36,37]**.** *Let* U *denote the set of arguments and* ρ(U) *denote the power set of* U*, given a set function:* χ:ρ(U)→[0,1] *if* χ *satisfies the following condition*:

(1) χ(∅)=0,χ(U)=1;
(2) ∀Y,Z∈ρ(U), if Y⊆Z,χ(Y)≤χ(Z).

Then, this will be considered a fuzzy measure.

λ-fuzzy measurements were first proposed by Sugeno; these satisfy the definition of fuzzy measurements while, at the same time, satisfying the following properties:

(2) μ(A∪B)=μ(A)+μ(B)+λμ(A)μ(B)

where λ∈(−1,+∞) and A∩B≠∅.

If X denotes a finite set, $\cup_{i=1}^{n}x_{i}=X$, λ−fuzzy measurement g satisfies the following equation:

(3) $$g\left(X\right)=\frac{1}{\lambda }\left(\overset{n}{\underset{i=1}{\prod }}\left(1+\lambda g\left(x_{i}\right)\right)-1\right)$$

When g(X)=1, the parameter λ can be determined by the following equation:

(4) $$\lambda +1=\overset{n}{\underset{i=1}{\prod }}\left(1+\lambda g\left(x_{i}\right)\right)$$

### 2.4. Prospect Theory

Kahneman and Tversky’s core idea in prospect theory is that investors’ decision-making behavior does not follow the expected utility maximization pattern but lies in the differences between the attribute value and the expected value. In the process of measuring the differences, instead of simply calculating the distance, the risk preference of decision-making is considered through the prospect function. Based on this idea, we restate the definition of prospect theory.

**Definition** **3** [38,39]**.** *The actual value of utility obtained by the decision-maker has a strong correlation with the distance from the prospect expectation. When the actual value is higher than expected, the utility that decision-makers obtain is greater than 0, i.e., it is beneficial. In terms of benefit, the attitude of decision-makers toward risk is preference. Conversely, when the actual value is lower than the expectation, the utility is lower than 0, and the attitude of decision-makers is aversion. Therefore, Kahneman and Tversky defined the value function as:*

(5) $$v\left(\Delta x_{ij}^{k}\right)=v\left(x_{ij}^{k}-p_{j}^{k}\right)=\{\begin{array}{c} {\left(\Delta x_{ij}^{k}\right)}^{\alpha },\Delta x_{ij}^{k}\geq 0\\ \lambda {\left(\Delta x_{ij}^{k}\right)}^{\beta },\Delta x_{ij}^{k}<0 \end{array}$$

*where* α *and* β *denote the gain and loss functions, respectively,* 0≤α,β≤1. λ *denotes the risk aversion coefficient when* λ>1*, indicating that the decision-maker is more sensitive to losses than to gains. After extensive experimentation, Kahneman and Tversky suggested that it is reasonable to set the parameters as* α=0.89, β=0.92, λ=2.25.

## 3. Framework of the Three-Way Approach Based on Non-Additive Measurements and Multiple-Source Incomplete Information Systems

In recent years, due to the continuously changing environment, decision-making information exhibits various characteristics, including incompleteness, ambiguity, and multi-source heterogeneity. In the process of decision-making, various characteristics exist in interactions and have a collaborative effect on the decision results. The main reason is that characteristics have non-independent relationships with each other. Accordingly, this paper adopted non-additive measurement to mine the non-independence characteristics of different attributes. Furthermore, individuals are emotional rather than economic beings, and the process of decision-making needs to consider their risk preferences. Prospect theory as a new tool describes the utility of decision-makers with a new function to map actual values to utility values. We utilize the utility function to improve the loss function of the original three-way decision-making model and propose an improved three-way decision-making model, as follows.

### 3.1. The Framework of MSIIS Based on Non-Additive Measurement and Prospect Theory

In this part, we define the framework of the proposed method, based on the above theoretical basis.

First, we define a multi-source incomplete information system.

**Definition** **4.** *Let the set of decision objects be:* $U=\left\{x_{1},x_{2},\ldots ,x_{m}\right\}$*, The set of attributes characterizing the object of the evaluation decision is:* $A=\left\{a_{1},a_{2},\ldots ,a_{n}\right\}$*, At the same time, there are* k *players involved in decision-making, corresponding to* k *decision-making information systems, according to which a binary relationship is established for each player* $f^{\left(k\right)}:U^{\left(k\right)}\times A^{\left(k\right)}$*,* $f^{\left(k\right)}\left(x_{i}^{k},a_{j}^{k}\right)$ *denotes the binary relationship between the decision object and its corresponding attribute, whereby the multi-source incomplete hybrid information system is composed* $\left(U,C_{k}\cup \left\{g_{k}\right\},A_{k},f_{k}\right)$*, in which* $C_{k}$ *represents the set of conditional attributes, and* $\left\{g_{k}\right\}$ *represents the set of decision attributes. The corresponding decision-making information system is shown in Table 1.*

(6) $$f_{ij}=\{\begin{array}{c} -,If\;the\;attribute\;information\;is\;unknown\\ r_{ij},If\;the\;attribute\;information\;is\;known \end{array}$$

The definition of an incomplete hybrid information system is given in Definition 4. How to measure an incomplete information system is an important issue when dealing with uncertain information. Therefore, we defined the measurement of distance in an incomplete information system.

**Definition** **5.** *In a multiple-source incomplete information system* $\left(U,C_{k}\cup \left\{g_{k}\right\},A_{k},f_{k}\right)$*, if the attribute value is Boolean, the formulas for calculating the distance between the attribute value and the utility are as follows:*

(7) $$d^{k}(x_{ij}^{k},\;p_{j}^{k})=\{\begin{array}{c} 0,\;x_{ij}^{k}=p_{j}^{k},\;or\;x_{ij}^{k}=*\\ 1,\;x_{ij}^{k}\neq p_{j}^{k} \end{array}$$

If the attribute value is a determinant, the formula for the distance between the attribute value and the utility is shown below:

(8) $$d^{k}(x_{ij}^{k},\;p_{j}^{k})=\{\begin{array}{c} 0,\;x_{ij}^{k}=p_{j}^{k},\;or\;x_{ij}^{k}=*\\ \left|x_{ij}^{k}-p_{j}^{k}\right|,\;x_{ij}^{k}\neq p_{j}^{k} \end{array}$$

If the attribute is an interval number, the formula for the distance between the attribute value and the utility is shown below:

(9) $$d^{k}(x_{ij}^{k},\;p_{j}^{k})=\{\begin{array}{c} 0,\;x_{ij}^{k}=p_{j}^{k},\;or\;x_{ij}^{k}=*\\ \sqrt{\frac{1}{2}\left[{\left(p_{j}^{k}-b_{ij}^{k}\right)}^{2}+{\left(p_{j}^{k}-a_{ij}^{k}\right)}^{2}\right]},\;x_{ij}^{k}\neq p_{j}^{k} \end{array}$$

**Property** **1.**

(1) $d^{k}\left(x_{ij}^{k},p_{j}^{k}\right)\leq 0,ifd^{k}\left(x_{ij}^{k},p_{j}^{k}\right)=0$*, meaning that the attribute value reaches the utility value, and the larger* $d^{k}\left(x_{ij}^{k},p_{j}^{k}\right)$ *is, the larger the gap between the attribute and utility, which leads to a larger loss for the decision-maker;*
(2) *When* $x_{ij}^{k}-p_{j}^{k}\geq 0$*, the attribute value is greater than the utility value, leading to gains. When* $x_{ij}^{k}-p_{j}^{k}<0$*, the attribute value is lower than the utility value, leading to losses.*

**Remark** **1.** *For the measurement of incomplete information, if the distance from utility is 0, its managerial meaning is that the impact of the missing value on the decision is irrelevant. If the initial value of incomplete information is set to 0, its managerial meaning is that the impact of the missing value on the decision is crucial, which is equivalent to adding a penalty factor. This incomplete information is an impediment to the decision-making methodology. The more incomplete the information is, the more hysteresis the sorting of decision-making plans will have, and the worse performance the attribute state will have.*

For incomplete information systems, solving attribute weight is a problem worth exploring. In Remark 1, two ways of incomplete information measurement are introduced. In terms of the influence of attribute weight, we expect that the more incomplete the information, the smaller the attribute weight, which will be converted to actual utility value. This paper uses the value matrix to solve attribute weight. For a value matrix corresponding to an incomplete information system, if the matrix is more widely dispersed, the larger the attribute weight is, and the more narrowly dispersed it is, the smaller the attribute weight is. Thus, the definition of information entropy is introduced, which was used as a measure of the attribute weight.

**Definition** **6.** *In a multiple-source incomplete information system* $\left(U,C_{k}\cup \left\{g_{k}\right\},A_{k},f_{k}\right)$*,* $U=\left\{x_{1},x_{2},\ldots ,x_{m}\right\}$*, the set of attribute features for evaluating the decision object is:* $A=\left\{a_{1},a_{2},\ldots ,a_{n}\right\}$; *at the same time, there are k players participating in decision-making.* $f^{\left(l\right)}\left(x_{i}^{l},a_{j}^{l}\right)$ *denotes the binary relationship between the decision object and its corresponding attribute.* $d^{k}\left(x_{ij}^{k},p_{j}^{k}\right)$ *denotes the distance between the attribute value and the utility value of the decision object, and* $v\left(C_{j}^{\left(\left(k\right)\right)}\right)$ *denotes the projection value of the distance with respect to the prospect theory, i.e., the actual utility that is obtained. Therefore, the value entropy of the decision attribute is defined as:*

(10) $$H\left(C_{j}^{\left(k\right)}\right)=-\overset{n}{\underset{i=1}{\sum }}v\left(C_{j}^{\left(k\right)}\right)\log_{2}v\left(C_{j}^{\left(k\right)}\right)$$

Based on the entropy value of each attribute, the method of defining the attribute weights is given as:

(11) $$w\left(C_{j}^{\left(k\right)}\right)=\frac{H\left(c_{j}^{\left(k\right)}\right)}{\sum_{k=1}^{K}\Sigma_{j=1}^{m_{k}}H\left(c_{j}^{\left(k\right)}\right)}=\frac{\sum_{i=1}^{n}v\left(C_{j}^{\left(k\right)}\right)\log_{2}v\left(C_{j}^{\left(k\right)}\right)}{\sum_{k=1}^{K}\Sigma_{j=1}^{m_{k}}\sum_{i=1}^{n}v\left(C_{j}^{\left(k\right)}\right)\log_{2}v\left(C_{j}^{\left(k\right)}\right)}$$

where $w\left(C_{j}^{\left(k\right)}\right)\in \left[0,1\right]$, and $\sum_{k=1}\sum_{j=1}w\left(C_{j}^{\left(k\right)}\right)=1$.

In the actual decision-making process, risk factors have interactions, and no single attribute can lead to accurate results. Considering the common role between the attributes and the non-additive measurement of the interaction of risk factors, a definition of the non-additive measurement of attributes was created. Thus, a fuzzy utility information system that considers the interaction of risk factors was established.

**Definition** **7.** *In the multiple-source incomplete information system* $\left(U,C_{k}\cup \left\{g_{k}\right\},A_{k},f_{k}\right)$*,* $U=\left\{x_{1},x_{2},\ldots ,x_{m}\right\}$*, the set of attribute characteristics of the evaluation decision object is:* $A=\left\{a_{1},a_{2},\ldots ,a_{n}\right\}$*. Meanwhile, there are k players involved in decision-making. Let* $A^{k}=\left\{C_{1}^{k},C_{2}^{k},\ldots ,C_{n_{k}}^{k}\right\}$. $C_{a}^{k}\subseteq A^{k}$ *denotes any non-empty subset of* $A^{k}$*. Accordingly, a fuzzy utility information system considering the interaction of risk factors was established as Table 2.*

**Definition** **8.** *In a multiple-source incomplete information system* $\left(U,C_{k}\cup \left\{g_{k}\right\},A_{k},f_{k}\right)$*, where* k ∈Φ*, let* $r^{\left(k\right)}\left(x_{i},A_{j}^{k}\right)$ *denote the degree of correlation deviation between* $x_{ij}^{\left(k\right)}$ *and* $A_{j}^{\left(k\right)}$*. Therefore, the correlation loss deviation of evaluation and utility is defined as:*

(12) $$r^{\left(k\right)}\left(x_{i},A_{j}^{k}\right)=\frac{U^{\left(k\right)}\left(x_{i},A_{j}^{k}\right)}{{max}_{i=1}^{n}U^{\left(k\right)}\left(x_{i},A_{j}^{k}\right)}$$

**Remark** **2.** *Correlation deviation* $r^{\left(k\right)}\left(x_{i},A_{j}^{k}\right)$ *denotes the correlation deviation of the attribute value from utility, which satisfies* $r^{\left(k\right)}\left(x_{i},A_{j}^{k}\right)\in \left[0,1\right]$.

**Definition** **9.** *In a multiple-source incomplete information system* $\left(U,C_{k}\cup \left\{g_{k}\right\},A_{k},f_{k}\right)$*, where* k ∈Φ*, let* $r^{\left(k\right)}\left(x_{i},A_{j}^{k}\right)$ *denotes the similarity between* $x_{ij}^{\left(k\right)}$ *and expectation* $p_{j}^{\left(k\right)}$*. Accordingly, for any two decision objects* $x_{m_{1}},x_{m_{2}}\in U$*, the cosine similarity is defined as:*

(13) $$Csr^{k}\left(x_{i_{1}},x_{i_{2}}\right)=\frac{\sum_{h\in \Gamma_{1}^{k}}\left(r^{\left(k\right)}\left(x_{i_{1}},A_{j}^{k}\right)\times r^{\left(k\right)}\left(x_{i_{2}},A_{j}^{k}\right)\right)}{\sqrt{\sum_{h\in \Gamma_{1}^{k}}{\left(r^{\left(k\right)}\left(x_{i_{1}},A_{j}^{k}\right)\right)}^{2}\times \sum_{h\in \Gamma_{1}^{k}}{\left(r^{\left(k\right)}\left(x_{i_{2}},A_{j}^{k}\right)\right)}^{2}}}$$

Furthermore, the δ-neighborhood similar class of the decision object $x_{i}$ is defined as:

(14) $$CSC_{\delta }^{k}\left(x_{i}\right)=\left\{x_{i}\in U|Csr^{k}\left(x_{i},x_{i_{2}}\right)\geq \delta ,x_{i_{2}}\in U\right\}$$

where 0 ≤δ≤1, denoting that when the similarity of the two decision objects is greater than or equal to δ, then the objects are similar with respect to thei attributes.

**Property** **2.** $Csr^{k}\left(x_{i_{1}},x_{i_{2}}\right)$ *denotes the cosine similarity between* $x_{i_{1}}$ *and* $x_{i_{2}}$*, which satisfies the following properties:*

(1) $Csr^{k}\left(x_{i_{1}},x_{i_{1}}\right)=1$*, denoting that the object itself has a cosine similarity of 1 to itself;*
(2) $Csr^{k}\left(x_{i_{1}},x_{i_{2}}\right)=Csr^{k}\left(x_{i_{2}},x_{i_{1}}\right)$*, denoting that the cosine similarity has symmetry.*

In the process of decision-making, the data-driven decision classification model is mainly based on objective data used to carry out the analysis, leading to strong objectivity when classifying decision-making information. Expert experience also has a great impact on decision-making because experts give their opinions based on their own knowledge and experience background, which has strong subjectivity and can involve misleading information. Therefore, to avoid personal subjectivity while combining the subjective opinions of people, this paper uses a data- and knowledge-driven model to categorize the decision objects. In the next section, we will describe the role of knowledge, where the expert gives direct information about the classification of the decision object by combining the existing data with his/her existing knowledge.

**Definition** **10.** *In a multiple-source incomplete information system* $\left(U,C_{k}\cup \left\{g_{k}\right\},V_{k},f_{k}\right)$*, where* k ∈Φ*, let* $\Omega^{k}=\left\{D_{A}^{k},D_{H}^{k},D_{R}^{k}\right\}$ *denote the set of states.* $V_{g_{k}}=\left\{A,H,R\right\}$ *denotes the value domain of the decision attribute* $g_{k}$*, whereby the three states of* $\Omega^{k}$ *are defined as:*

(15) $$\begin{array}{l} D_{A}^{k}=\left\{x_{j}\in U|g_{ik}=A\right\}\\ D_{H}^{k}=\left\{x_{j}\in U|g_{ik}=H\right\}\\ D_{R}^{k}=\left\{x_{j}\in U|g_{ik}=R\right\} \end{array}$$

*where *$D_{A}^{k}$* indicates that the state of the decision object is in a good state, and the corresponding measurements need to be taken. This situation is considered as acceptance of the decision; meanwhile, it belongs to the critical decision object. From the point of view of risk analysis, compared with the other two situations, this situation is the purview of the experts, based on the experiences they have already had, and the analysis judges that this failure mode is a critical failure mode, and the probability of occurrence of that failure is extremely high. *$D_{H}^{k}$* denotes the set of decision objects for which there is no judgment on whether there is a risk or not, where the expert may not be able to give an accurate decision based on their existing empirical knowledge. Therefore, the expert chooses to delay the decision, thus defining it as an object set waiting for classification or a delayed decision.*

For the three different states, three targeted decisions correspond to them at the same time, i.e., $\beta =\left\{b_{A},b_{H},b_{R}\right\}$, where $b_{A}$ denotes the acceptance decision, $b_{H}$ denotes the delay decision, and $b_{R}$ denotes the rejection decision. Accordingly, the loss function matrix of the three states and three decisions corresponding to each object can be established as shown in Table 3.

**Definition** **11.** *In a multiple-source incomplete information system* $\left(U,C_{k}\cup \left\{g_{k}\right\},A_{k},f_{k}\right)$*, where* k ∈Φ*, given a set function* ρ:P(U)→[0,1]*. Let* $CSC_{\delta }^{k}\left(x_{i}\right)$ *denote the* δ*-cosine similar class. For a situation where* X∈P(U)*, the probability measurement of object* X *is defined as:*

(16) $$\rho \left(X\right)=\frac{\left|\left|\left\{x_{i}\in U|CSC_{\delta }^{k}\left(x_{i}\right)\cap X\neq \emptyset \right\}\right|\right|}{\left|U\right|}$$

**Definition** **12.** *In a multiple-source incomplete information system* $\left(U,C_{k}\cup \left\{g_{k}\right\},A_{k},f_{k}\right)$*, where* k∈Φ*, given a set function* ρ:P(U)→[0,1]*. Let* $\Omega^{k}=\left\{D_{A}^{k},D_{H}^{k},D_{R}^{k}\right\}$ *denote the set of states. Thus, the method of calculating the relevance of* $x_{i}$*, belonging to the state* $D_{⋄}^{k}\left(⋄\in \left\{A,H,R\right\}\right),$ *is defined as:*

(17) $$P_{N}\left(D_{⋄}^{k}|x_{i}\right)=\frac{\rho \left(\left\{x_{i}\right\}\right)}{\rho \left(\left\{x_{i}\right\}\right)+\rho \left(D_{⋄}^{k}\right)}$$

### 3.2. The Three-Way Decision Framework Based on Non-Additive Measurements and the Approach of Fusion Information

In this section, we combine the method of calculating the loss function via the prospect theory proposed in Section 2.4 to propose a new dynamic three-way consensus decision model. Firstly, we give the calculation of the expected loss:

(18) $$L_{P}^{k}\left(b_{P}|x_{i}\right)=l_{PB}^{i}P_{N}\left(B|x_{i}\right)+l_{PN}^{i}P_{N}\left(N|x_{i}\right)\;L_{B}^{k}\left(b_{B}|x_{i}\right)=l_{BP}^{i}P_{N}\left(P|x_{i}\right)+l_{BN}^{i}P_{N}\left(N|x_{i}\right)\;L_{N}^{k}\left(b_{N}|x_{i}\right)=l_{NP}^{i}P_{N}\left(P|x_{i}\right)+l_{NB}^{i}P_{N}\left(B|x_{i}\right)$$

Six correlation loss functions were calculated:

(19) $$l_{BP}^{i}=\mu \left(1-\underset{h_{1}\in \Gamma_{1}^{k}}{\sum }\left(w_{h}r^{\left(l\right)}\left(x_{ih_{1}}^{\left(l\right)},p_{h_{1}}^{\left(l\right)}\right)\right)-\underset{h_{2}\in \Gamma_{2}^{k}}{\sum }\left(w_{h}r^{\left(l\right)}\left(x_{ih_{2}}^{\left(l\right)},p_{h_{2}}^{\left(l\right)}\right)\right)\right)\;l_{PB}^{i}=v\left(\underset{h_{1}\in \Gamma_{1}^{k}}{\sum }\left(w_{h}r^{\left(l\right)}\left(x_{ih_{1}}^{\left(l\right)},p_{h_{1}}^{\left(l\right)}\right)\right)+\underset{h_{2}\in \Gamma_{2}^{k}}{\sum }\left(w_{h}r^{\left(l\right)}\left(x_{ih_{2}}^{\left(l\right)},p_{h_{2}}^{\left(l\right)}\right)\right)\right)\;l_{NP}^{i}=1-\underset{h_{1}\in \Gamma_{1}^{k}}{\sum }\left(w_{h}r^{\left(l\right)}\left(x_{ih_{1}}^{\left(l\right)},p_{h_{1}}^{\left(l\right)}\right)\right)-\underset{h_{2}\in \Gamma_{2}^{k}}{\sum }\left(w_{h}r^{\left(l\right)}\left(x_{ih_{2}}^{\left(l\right)},p_{h_{2}}^{\left(l\right)}\right)\right)\;l_{NB}^{i}=v\left(1-\underset{h_{1}\in \Gamma_{1}^{k}}{\sum }\left(w_{h}r^{\left(l\right)}\left(x_{ih_{1}}^{\left(l\right)},p_{h_{1}}^{\left(l\right)}\right)\right)-\underset{h_{2}\in \Gamma_{2}^{k}}{\sum }\left(w_{h}r^{\left(l\right)}\left(x_{ih_{2}}^{\left(l\right)},p_{h_{2}}^{\left(l\right)}\right)\right)\right)\;l_{PN}^{i}=\underset{h_{1}\in \Gamma_{1}^{k}}{\sum }\left(w_{h}r^{\left(l\right)}\left(x_{ih_{1}}^{\left(l\right)},p_{h_{1}}^{\left(l\right)}\right)\right)+\underset{h_{2}\in \Gamma_{2}^{k}}{\sum }\left(w_{h}r^{\left(l\right)}\left(x_{ih_{2}}^{\left(l\right)},p_{h_{2}}^{\left(l\right)}\right)\right)\;l_{BN}^{i}=\mu \left(\underset{h_{1}\in \Gamma_{1}^{k}}{\sum }\left(w_{h}r^{\left(l\right)}\left(x_{ih_{1}}^{\left(l\right)},p_{h_{1}}^{\left(l\right)}\right)\right)+\underset{h_{2}\in \Gamma_{2}^{k}}{\sum }\left(w_{h}r^{\left(l\right)}\left(x_{ih_{2}}^{\left(l\right)},p_{h_{2}}^{\left(l\right)}\right)\right)\right)$$

where μ∈(0,1) and v∈(0,1).

(P) has $x_{i}\in Pos\left(D_{P}^{k}\right)$, if $L_{B}^{k}\left(b_{P}|x_{i}\right)\leq L_{P}^{k}\left(b_{B}|x_{i}\right)$ and $L_{B}^{k}\left(b_{P}|x_{i}\right)\leq L_{N}^{k}\left(b_{N}|x_{i}\right)$;

(B) has $x_{i}\in Bnd\left(D_{P}^{k}\right)$, if $L_{B}^{k}\left(b_{B}|x_{i}\right)\leq L_{P}^{k}\left(b_{P}|x_{i}\right)$ and $L_{B}^{k}\left(b_{B}|x_{i}\right)\leq L_{N}^{k}\left(b_{N}|x_{i}\right)$;

(N) has $x_{i}\in Neg\left(D_{P}^{k}\right)$, if $L_{N}^{k}\left(b_{N}|x_{i}\right)\leq L_{P}^{k}\left(b_{P}|x_{i}\right)$ and $L_{N}^{k}\left(b_{N}|x_{i}\right)\leq L_{B}^{k}\left(b_{B}|x_{i}\right)$.

(P1) has $x_{i}\in Pos\left(D_{P}^{k}\right)$, if $P_{N}\left(D_{P}^{k}|x_{i}\right)\geq \alpha_{i}^{k}$ and $P_{N}\left(D_{P}^{k}|x_{i}\right)\geq \gamma_{i}^{k}$;

(B1) has $x_{i}\in Bnd\left(D_{P}^{k}\right)$, if $P_{N}\left(D_{P}^{k}|x_{i}\right)\leq \alpha_{i}^{k}$ and $P_{N}\left(D_{P}^{k}|x_{i}\right)\geq \beta_{i}^{k}$;

(N1) has $x_{i}\in Neg\left(D_{P}^{k}\right)$, if $P_{N}\left(D_{P}^{k}|x_{i}\right)\leq \gamma_{i}^{k}$ and $P_{N}\left(D_{P}^{k}|x_{i}\right)\leq \beta_{i}^{k}$.

Accordingly, the calculation of the threshold is given as shown below:

(20) $$\alpha_{i}^{k}=\frac{l_{PN}^{i}-l_{BN}^{i}}{\left(l_{PN}^{i}-l_{BN}^{i}\right)+l_{BP}^{i}}\;\gamma_{i}^{k}=\frac{l_{PN}^{i}}{l_{PN}^{i}+l_{NP}^{i}}\;\beta_{i}^{k}=\frac{l_{BN}^{i}}{l_{BN}^{i}+\left(l_{NP}^{i}-l_{BP}^{i}\right)}$$

Considering that different decision-makers will approach a problem with different risk preferences, this paper divides the results by optimism, pessimism, and variable precision and gives them definitions.

**Definition** **13.** *Let* $\left(U,C_{k}\cup \left\{g_{k}\right\},A_{k},f_{k}\right)$ *be an incomplete information system, where* $U^{\left(l\right)}$ *denotes a subset of decision objects and* $A^{\left(l\right)}$ *denotes a subset of attributes. For any* $P^{\left(l\right)}\in F\left(V\right)$ *and* $x^{\left(l\right)}\in U^{\left(l\right)}$*, the positive and negative domains in an optimistic case for incomplete information systems are defined as:*

(21) $$Pos^{O}\left(D_{P}\right)=\cup Pos\left(D_{P}^{k}\right)\;Neg^{O}\left(D_{P}\right)=\cap Neg\left(D_{P}^{k}\right)\;Bun^{O}\left(D_{P}\right)=U-Pos^{O}\left(D_{P}\right)-Neg^{o}\left(D_{P}\right)\;\;\;\;\;\;\;\;\;\;\;\;\;\;\;\;\;\;\;\;\;\;\;\;\;\;\;\;\;\;\;\;\;\;\;\;=U-\cup Pos\left(D_{P}^{k}\right)-\cap Neg\left(D_{P}^{k}\right)$$

**Definition** **14.** *Let* $\left(U,C_{k}\cup \left\{g_{k}\right\},A_{k},f_{k}\right)$ *be an incomplete information system, where* $U^{\left(l\right)}$ *denotes a subset of decision objects and* $A^{\left(l\right)}$ *denotes a subset of attributes. For any* $P^{\left(l\right)}\in F\left(V\right)$ *and* $x^{\left(l\right)}\in U^{\left(l\right)}$*, the positive and negative domains in a pessimistic case for incomplete information systems are defined as:*

(22) $$Pos^{P}\left(D_{P}\right)=\cap Pos\left(D_{P}^{k}\right)\;Neg^{P}\left(D_{P}\right)=\cup Neg\left(D_{P}^{k}\right)\;Bun^{P}\left(D_{P}\right)=U-Pos^{P}\left(D_{P}\right)-Neg^{P}\left(D_{P}\right)\;\;\;\;\;\;\;\;\;\;\;\;\;\;\;\;\;\;\;\;\;\;\;\;\;\;\;\;\;\;\;\;\;\;\;\;=U-\cap Pos\left(D_{P}^{k}\right)-\cup Neg\left(D_{P}^{k}\right)$$

The two risk preferences, optimistic and pessimistic, are so extreme that in practical problems, there is a possibility that there is no solution. To make the conditions looser, the concept of variable precision is employed. Before that, it is necessary to define the signal function in both the positive and negative domains:

(23) $$sig^{\left(k\right)}\left(\left\{x_{i}|x_{i}\in Pos\left(D_{P}^{k}\right)\right\}\right)=\{\begin{array}{c} 0,\;x_{i}\notin Pos\left(D_{P}^{k}\right)\\ 1,x_{i}\in Pos\left(D_{P}^{k}\right) \end{array}\;sig^{\left(k\right)}\left(\left\{x_{i}|x_{i}\in Pos\left(D_{P}^{k}\right)\right\}\right)=\{\begin{array}{c} 0,\;x_{i}\notin Neg\left(D_{P}^{k}\right)\\ 1,x_{i}\in Neg\left(D_{P}^{k}\right) \end{array}$$

**Definition** **15.** *Let* $\left(U,C_{k}\cup \left\{g_{k}\right\},A_{k},f_{k}\right)$ *be an incomplete information system,* $U^{\left(l\right)}$ *denotes a subset of decision objects and* $A^{\left(l\right)}$ *denotes a subset of attributes. For any* $P^{\left(l\right)}\in F\left(V\right)$ *and* $x^{\left(l\right)}\in U^{\left(l\right)}$*, (I) variable accuracy is present in the system of positive and negative domains (I)* $-\left(\zeta_{1},\zeta_{2}\right)$*:*

(24) $$(I)\;Pos^{\zeta_{1}}\left(D_{P}\right)=\left\{x_{i}|\frac{\sum_{k}sig^{\left(k\right)}\left(\left\{x_{i}|x_{i}\in Pos\left(D_{P}^{k}\right)\right\}\right)}{\left|U\right|}\geq \zeta_{1}\right\}\;(I)\;Neg^{\zeta_{2}}\left(D_{P}\right)=\left\{x_{i}|\frac{\sum_{k}sig^{\left(k\right)}\left(\left\{x_{i}|x_{i}\in Neg\left(D_{P}^{k}\right)\right\}\right)}{\left|U\right|}\leq \zeta_{2}\;\right\}\;(I)\;Bun^{\left(\zeta_{1},\zeta_{2}\right)}\left(D_{P}\right)=U-\left(I\right)Pos^{\zeta_{1}}\left(D_{P}\right)-\left(I\right)Neg^{\zeta_{2}}\left(D_{P}\right)=U-\;\left\{x_{i}|\frac{\sum_{k}sig^{\left(k\right)}\left(\left\{x_{i}|x_{i}\in Pos\left(D_{P}^{k}\right)\right\}\right)}{\left|U\right|}\geq \zeta_{1}\;\right\}-\left\{x_{i}|\frac{\sum_{k}sig^{\left(k\right)}\left(\left\{x_{i}|x_{i}\in Neg\left(D_{P}^{k}\right)\right\}\right)}{\left|U\right|}\leq \zeta_{2}\;\right\}$$

*(II) variable accuracy is present in the system of positive and negative domains (II)* $-\left(\zeta_{1},\zeta_{2}\right)$*:*

(25) $$(II)\;Pos^{\zeta_{1}}\left(D_{P}\right)=\left\{x_{i}|\frac{\sum_{k}sig^{\left(k\right)}\left(\left\{x_{i}|x_{i}\in Pos\left(D_{P}^{k}\right)\right\}\right)}{\left|U\right|}\leq \zeta_{1}\;\;\right\}\;(II)\;Neg^{\zeta_{2}}\left(D_{P}\right)=\left\{x_{i}|\frac{\sum_{k}sig^{\left(k\right)}\left(\left\{x_{i}|x_{i}\in Neg\left(D_{P}^{k}\right)\right\}\right)}{\left|U\right|}\geq \zeta_{2}\;\right\}\;(II)\;Bun^{\left(\zeta_{1},\zeta_{2}\right)}\left(D_{P}\right)=U-\left(II\right)Pos^{\zeta_{1}}\left(D_{P}\right)-\left(II\right)Neg^{\zeta_{2}}\left(D_{P}\right)=U-\;\left\{x_{i}|\frac{\sum_{k}sig^{\left(k\right)}\left(\left\{x_{i}|x_{i}\in Pos\left(D_{P}^{k}\right)\right\}\right)}{\left|U\right|}\leq \zeta_{1}\;\right\}-\left\{x_{i}|\;\frac{\sum_{k}sig^{\left(k\right)}\left(\left\{x_{i}|x_{i}\in Neg\left(D_{P}^{k}\right)\right\}\right)}{\left|U\right|}\geq \zeta_{2}\right\}$$

**Theorem** **1.**

(1) *When* $\zeta_{1}=1,\zeta_{2}=\frac{1}{\left|U\right|}$*, the (I) variable precision model degenerates into a classification result of the pessimistic case.*
(2) *When* $\zeta_{1}=\frac{1}{\left|U\right|},\zeta_{2}=1$*, the (II) variable precision model degenerates into a classification result of the optimistic case.*

**Proof:**  (1) When $\zeta_{1}=1,\zeta_{2}=\frac{1}{\left|U\right|}$, there is:

(26) $$Pos^{\zeta_{1}}\left(D_{P}\right)=\left\{x_{i}|\frac{\sum_{k}sig^{\left(k\right)}\left(\left\{x_{i}|x_{i}\in Pos\left(D_{P}^{k}\right)\right\}\right)}{\left|U\right|}\geq \zeta_{1}\;\right\}\;\;\;\;\;\;\;=\left\{x_{i}|\frac{\sum_{k}sig^{\left(k\right)}\left(\left\{x_{i}|x_{i}\in Pos\left(D_{P}^{k}\right)\right\}\right)}{\left|U\right|}\geq 1\;\right\}$$

At this point,

(27) $$\frac{\sum_{k}sig^{\left(k\right)}\left(\left\{x_{i}|x_{i}\in Pos\left(D_{P}^{k}\right)\right\}\right)}{\left|U\right|}=1$$

i.e.:

(28) $$\underset{k}{\sum }sig^{\left(k\right)}\left(\left\{x_{i}|x_{i}\in Pos\left(D_{P}^{k}\right)\right\}\right)=\left|U\right|$$

For decision object $x_{i}$:

(29) $$x_{i}\in Pos\left(D_{P}^{1}\right)\cap x_{i}\in Pos\left(D_{P}^{2}\right)\cap \cdots \cap x_{i}\in Pos\left(D_{P}^{k}\right),$$

and, thus, $Pos^{P}\left(D_{P}\right)=\cap Pos\left(D_{P}^{k}\right)$ is proved.

(30) $$Neg^{\zeta_{1}}\left(D_{P}\right)=\left\{x_{i}|\frac{\sum_{k}sig^{\left(k\right)}\left(\left\{x_{i}|x_{i}\in Neg\left(D_{P}^{k}\right)\right\}\right)}{\left|U\right|}\leq \zeta_{1}\;\right\}\;\;\;\;\;\;\;\;\;=\left\{x_{i}|\frac{\sum_{k}sig^{\left(k\right)}\left(\left\{x_{i}|x_{i}\in Pos\left(D_{P}^{k}\right)\right\}\right)}{\left|U\right|}\leq \frac{1}{\left|U\right|}\;\right\}$$

At this point,

(31) $$\frac{\sum_{k}sig^{\left(k\right)}\left(\left\{x_{i}|x_{i}\in Neg\left(D_{P}^{k}\right)\right\}\right)}{\left|U\right|}=\frac{1}{\left|U\right|}$$

i.e.:

(32) $$\underset{k}{\sum }sig^{\left(k\right)}\left(\left\{x_{i}|x_{i}\in Neg\left(D_{P}^{k}\right)\right\}\right)=1$$

For decision object $x_{i}$:

(33) $$x_{i}\in Neg\left(D_{P}^{1}\right)\cup x_{i}\in Neg\left(D_{P}^{2}\right)\cup \cdots \cup x_{i}\in Neg\left(D_{P}^{k}\right)$$

In the same way, (2) is proved. □

## 4. A New Risk Analysis Method for a Three-way Decision-making Model Based on Prospect Theory and Non-Additive Measurements

For risk analysis issues, most of the existing studies focus on analyzing the potential risks from a single information source [31,32,40,41]. However, in today’s complex and volatile decision-making environment, the key risk factors identified from one information source can no longer meet the demands for analyzing, assessing, and predicting equipment safety risks under realistic conditions. Therefore, this article comprehensively evaluates equipment in-service risks by fusing the risk information from three aspects: inherited risk analysis results from the design phase, safety events recorded during its service, and real-time operation data or simulation data concerning the real-world environment. Therefore, a new 3WD risk analysis method is proposed to deal with such circumstances. The main risk analysis issue involved is described in the first part of this section. Then, the procedure for this method is illustrated.

### 4.1. Problem Statement

In this section, we introduce the multi-granularity three-way decision-making risk analysis model. We analyze the existing risk data from three aspects:

(1) For the design stage, we collected original FMEA results.
(2) For the in-service stage, we collected equipment safety reports, including records of accidents, incidents, failures, etc.
(3) For the simulation experiment, we collected the response data of equipment modeling under the virtual environment.

Thus, the risk factors were analyzed by combining the three aspects of information on the aviation equipment, which were presented in the form of multi-granularity. Finding a way to integrate the three aspects of granularity of the information is a very meaningful research approach. This paper improved the FMEA method, so as to put forward a new FMEA risk analysis framework, rather than presenting a simple analysis of the initial information since actual risk analysis is a dynamic process.

For the proposed new FMEA model, this paper focuses on analyzing the criticality of the different failure modes by combining three aspects of information. In terms of the granularity of the information, the different failure modes that are obtained vary. The obtained multi-granularity hybrid information system is shown in Table 4.

For the proposed new FMEA risk analysis method, this paper focuses on how to integrate information from three different granularity aspects to obtain dynamic critical failure modes that change continuously with the information, and, ultimately, how to assess and analyze safety risk.

### 4.2. Description of the Three-Way Risk Analysis Method

Working according to the new model proposed in Section 4.1, the risk analysis model, which is based on fuzzy measurement and dynamic three-way consensus decision-making, is re-explained in this section and its algorithmic flow is shown as follows:

Step 1: Obtaining data from three aspects, comprising the FMEA results at the design stage, the statistical analysis of the actual event occurrence, and the simulation’s experimental data.

Step 2: Integrating the information from these three aspects to obtain three granularities for analyzing the different failure modes and assigning different weights to the attribute information of the three aspects: $\lambda =\left\{\lambda_{1},\lambda_{2},\ldots ,\lambda_{l}\right\}$.

Step 3: An incomplete information system is obtained, a suitable weight determination method is selected for the incomplete information system, and the attribute weights are determined: $w=\left\{w_{1},w_{2},\ldots ,w_{n}\right\}$.

Step 4: Using non-additive measurements, the three-way decision-making model under each attribute is built with its corresponding loss function; the reference point is determined first, and the gain-loss matrix is obtained:

(34) $$G^{\left(l\right)}\left(x_{ij}^{\left(l\right)},p_{j}^{\left(l\right)}\right)=\{\begin{array}{c} -d^{\left(l\right)}\left(x_{ij}^{\left(l\right)},p_{j}^{\left(l\right)}\right),x_{ij}^{\left(l\right)}<p_{j}^{\left(l\right)}\\ d^{\left(l\right)}\left(x_{ij}^{\left(l\right)},p_{j}^{\left(l\right)}\right),x_{ij}^{\left(l\right)}\geq p_{j}^{\left(l\right)} \end{array}$$

Step 5: Based on the prospective utility function, the gain-loss matrix is mapped using the prospective utility function to obtain the true loss value:

(35) $$v\left(x_{i},x_{s}\right)=\{\begin{array}{c} -G^{\alpha },x\geq 0\\ \lambda {\left(-G\right)}^{\beta },x<0 \end{array}$$

Calculating the value of loss considering the risk appetite of the decision-maker is shown in Table 5.

Considering the non-independence seen between the attributes, the Choquet integral is used to fuse the expectations of different attributes. A decision rule is established based on the loss function so as to triple the failure modes, whereby the set of objects for the critical risk mode, the medium risk mode, and the low-risk mode is given as shown in Table 6.

Step 6: Based on the combination of critical-risk mode, medium-risk mode, and low-risk mode for each attribute, the information is fused using rough sets to obtain the final set of risk factors, with $Pos\left(D_{P}^{k}\right)$ denoting the acceptance of the failure mode, $Neg\left(D_{P}^{k}\right)$ denoting the rejection of the failure mode, and $Bnd\left(D_{P}^{k}\right)$ denoting the delayed decision. The fusion of failure modes at different levels of granularity is carried out.

Step 7: By determining $\frac{\left|Bnd\left(D_{P}^{k}\right)\right|}{\left|U\right|}\leq \theta$, if the condition is satisfied, the division set is the final division set, which is the final classification result. Thus, the key failure mode set is obtained. If the condition is not satisfied, Step 6 is repeated to re-analyze and evaluate the failure modes to reach a consensus, and to reduce the number of decision-making objects in the intermediate boundary domain. Moreover, the division is clearer, and the optimal scheme preference criterion can be obtained.

**Figure 1:**
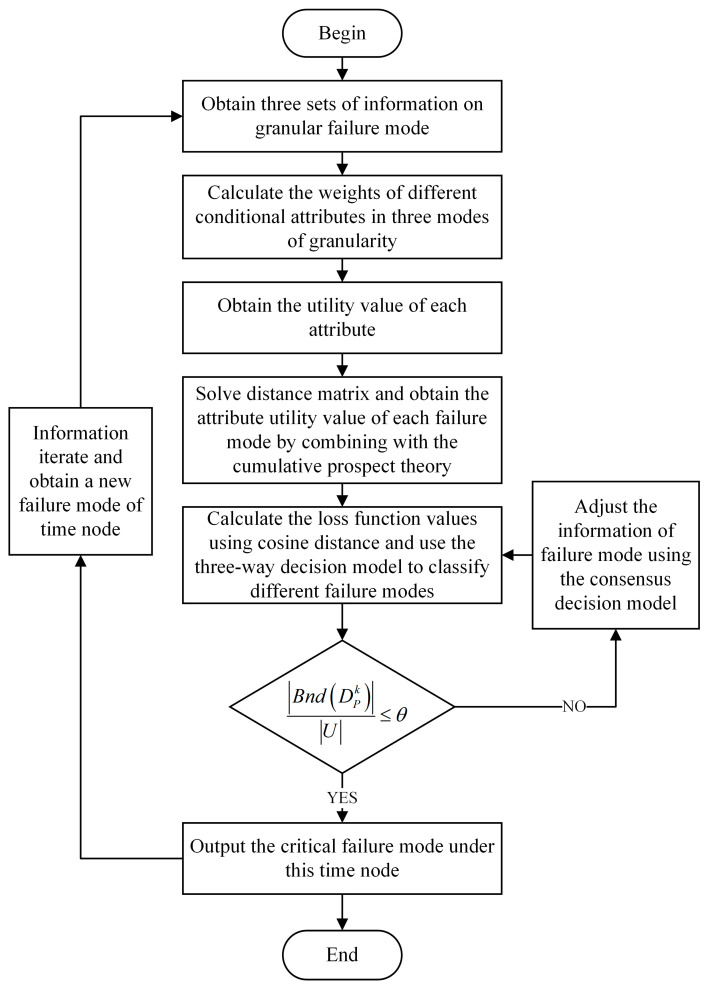
The flow of the proposed method.

When considering risk analysis in different scenarios and taking into account changes over time, one must bear in mind that the performance of critical parts in aviation equipment will constantly change and iterate during the occurrence of new unsafe events. Based on this evolution, in the next section, combined with the dynamic three-way decision-making model, this paper establishes a time-sequential dynamic three-way FMEA risk analysis model, as shown in Figure 1; the algorithm is shown as Algorithm 1.
**Algorithm 1:** The algorithm of the three-way decision-making approach
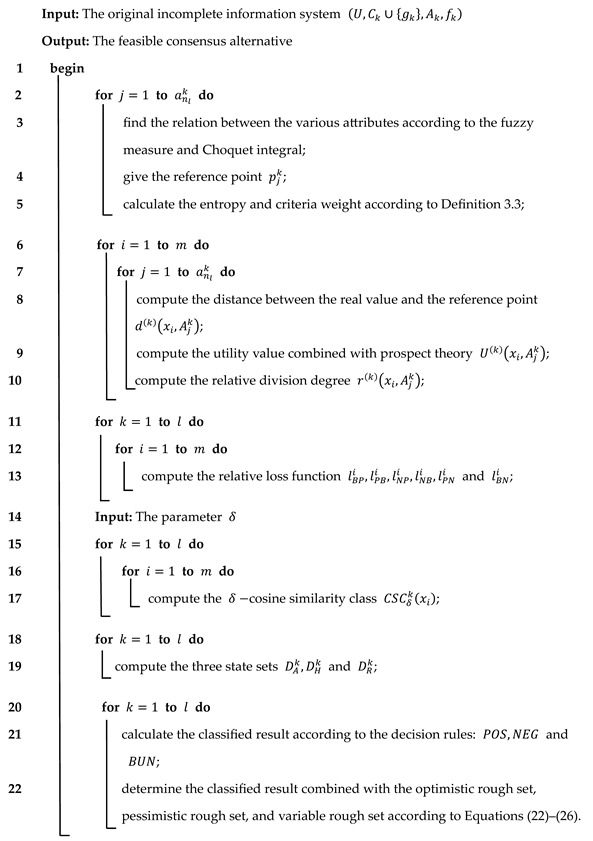


## 5. Example and Simulation Analysis

For this section, we carried out an example and simulation analysis. For the risk evaluation problem of in-service aircraft structures, we collected risk information on a civil aircraft from three sources: the first is the FMEA results from the aircraft design stage, which recorded the aircraft structure component failure modes, with the corresponding occurrence level and severity level; the second is the mechanical simulation results of aircraft structural components, where the relevant static force index and fatigue life index are collected; the third is the historical unsafe events statistical analysis results, which determine the event cause, i.e., the culprit structure components, along with their problem frequency and influence index.

Based on the above risk information and indexes, the incomplete information system was established as in Table 7. According to the illustrated processing steps and algorithm, the established incomplete information system is dealt with.

The preliminary results of the key factors from the three different scenarios are obtained as follows:

$POS^{1}=\left\{A_{8},A_{9},A_{10}\right\}$,

$BUN^{1}=\left\{A_{1}\right\}$,

$NEG^{1}=\left\{A_{2},A_{3},A_{4},A_{5},A_{6},A_{7},A_{11}\right\}$,

$POS^{2}=\left\{A_{10},A_{11}\right\}$,

$BUN^{2}=\left\{A_{6}\right\}$,

$NEG^{2}=\left\{A_{1},A_{2},A_{3},A_{4},A_{5},A_{7},A_{8},A_{9}\right\}$,

$POS^{3}=\left\{A_{10}\right\}$,

$BUN^{3}=\left\{A_{1},A_{2},A_{3},A_{4},A_{7}\right\}$,

$NEG^{3}=\left\{A_{5},A_{6},A_{8},A_{9},A_{11}\right\}$.

The preliminary results of the key factors from the three different scenarios are obtained as shown in Table 8.

According to Definition 13, it can be seen that the critical parts screened out in the optimistic scenario are as follows:

$POS^{O}=\left\{A_{2},A_{3},A_{5}\right\}$

$NEG^{O}=\left\{A_{6},A_{7},A_{8},A_{9},A_{11}\right\}$

$BUN^{O}=\left\{A_{1},A_{4},A_{10}\right\}$.

**Figure 2:**
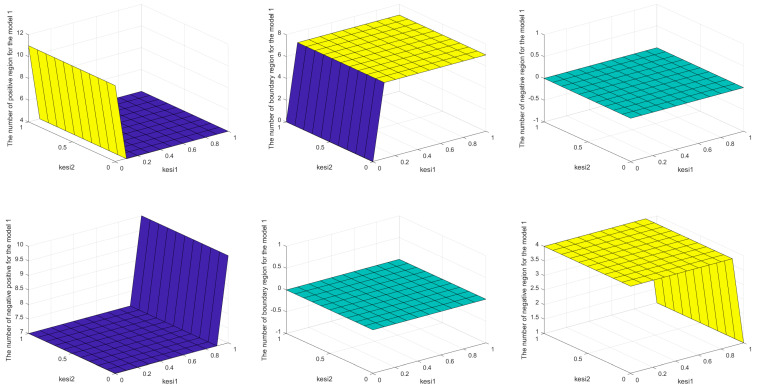
The simulation results of parameters $\zeta_{1}$ and $\zeta_{2}$.

In order to find the optimal granularity, we can seek the optimal parameters $\zeta_{1}$ and $\zeta_{2}$ by simulation analysis; the results are shown in Figure 2.

The first aspect is the loss function. TOPSIS is an objective decision-making model that evaluates the superiority of solutions. It identifies ideal and non-ideal points from the available data and calculates the distance between each decision object and the positive and negative ideals. Decision-makers prefer solutions that are closer to the positive ideal and further away from the negative ideal. However, this algorithm has its limitations. The results are relatively objective and it fails to consider the limited rationality of decision-makers. Additionally, when there is an excessive amount of data on decision objects, the discrimination ability of the algorithm gradually decreases, especially causing trouble when defining the weights of attribute information, which can undermine the accuracy of the algorithm. 

Clustering algorithms, on the other hand, aim to categorize decision objects with high similarity into the same group, with high similarity within a cluster and low similarity between clusters, thus classifying decision objects more accurately. Although existing clustering algorithms are mature, they also have their shortcomings. Similarly, they exhibit high objectivity. Given the classification characteristics of our solutions and the level of human cognition regarding different issues, we hope to obtain three types of categorical classifications for decision objects. However, when k = 3, the uncertainty of the classification results for decision objects using the three clustering algorithms is relatively high, which can affect the classification outcomes.

Ye et al. [27] proposed a new three-way group decision-making model by combining three-way decision-making with the preference-approval structure. This new model improved the preference-approval model based on the three-way decision-making framework. However, its limitation lies in that when the number of decision objects exceeds 10, the complexity of the algorithm increases significantly, making it difficult to obtain results, thus rendering it unsuitable for scenarios with more than 10 decision objects.

**Figure 3:**
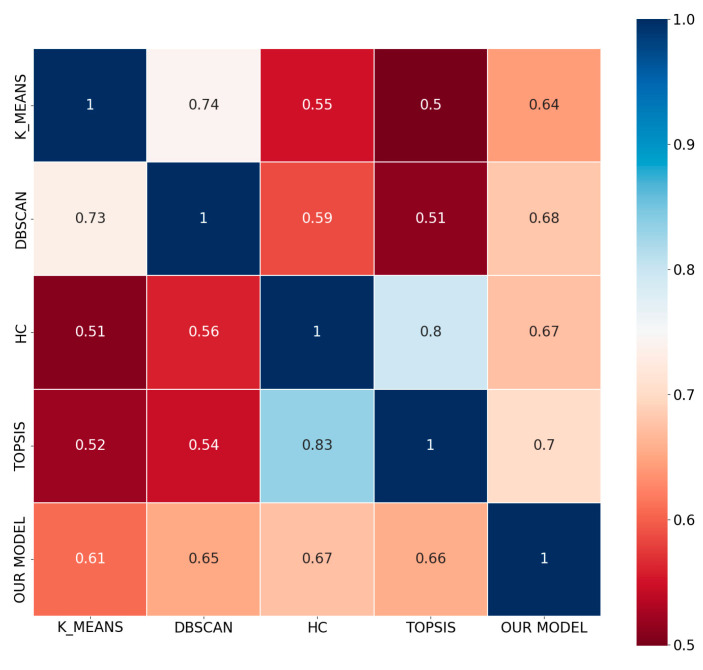
The similarity of the various methods.

Our newly proposed method can avoid the aforementioned issues as shown in Table 9. As seen from the comparison of algorithm similarity shown in Figure 3, the four objective methods exhibit a relatively high degree of similarity, while there is still a certain difference in terms of similarity between the methods proposed by Ye and our new approach. This aspect needs to be improved and is an area where our algorithm can be optimized further.

## 6. Conclusions

This paper considers the interactive relationship between risk factors, which leads to the joint influence of different factors on risk analysis. Meanwhile, by considering the different risk attitudes of decision-makers, combined non-additive measurements and the cumulative prospect theory were utilized to improve and propose a new solution for loss function. Based on this new solution, a three-way decision-making model was proposed after improvement. When faced with a variable and complex mission environment, by combining information from different stages of the equipment’s lifecycle, a new risk analysis model based on non-additive measurement and dynamic three-way consensus decision-making was proposed. The proposed model was applied to the safety risk problem of aviation equipment to ascertain critical failure mode and verify effectiveness. Through a comparison of different algorithms, the advantage that our proposed method can offer to handle a situation where the decision objects number more than 10 was proved.

The main innovations of this paper lie in three aspects. Firstly, the problem of how to solve the loss function is improved by considering the interactions between different factors. Secondly, a dynamic three-way decision-making model and the risk analysis model are combined by considering information on the three aspects to propose a new risk analysis model based on non-additive measurement and dynamic three-way decision-making. Lastly, the proposed model was applied to a safety risk analysis of aviation equipment, which provides strong theoretical support for its use in practical problems. The strengths of the proposed model lie in the interactivity of factors, which enables more comprehensive risk analysis, and the integration of equipment lifecycle information, which enhances the accuracy of the risk analysis. However, the shortcomings of this model are its complexity, which increases the difficulty of understanding it and its appropriate application, and its generalizability for different fields or types of risk analysis needs further verification.

## Figures and Tables

**Table 1:** The incomplete decision information system.

R	$e_{1}$			…	$e_{l}$			$g_{k}$
	$a_{1}^{1}$	…	$a_{n_{1}}^{1}$	…	$a_{1}^{l}$	…	$a_{n_{1}}^{l}$	
$x_{1}$	$f^{1}\left(x_{1},a_{1}^{1}\right)$	…	$f^{1}\left(x_{1},a_{n_{1}}^{1}\right)$	…	$f^{l}\left(x_{1},a_{1}^{1}\right)$	…	$f^{l}\left(x_{1},a_{n_{1}}^{l}\right)$	$g_{1}$
$x_{2}$	$f^{1}\left(x_{2},a_{1}^{1}\right)$	…	$f^{1}\left(x_{2},a_{n_{1}}^{1}\right)$	…	$f^{l}\left(x_{2},a_{1}^{1}\right)$	…	$f^{l}\left(x_{2},a_{n_{1}}^{l}\right)$	$g_{2}$
…	…	…	…	…	…	…	…	…
$x_{m}$	$f^{1}\left(x_{m},a_{1}^{1}\right)$	…	$f^{1}\left(x_{m},a_{n_{1}}^{1}\right)$	…	$f^{l}\left(x_{m},a_{1}^{1}\right)$	…	$f^{l}\left(x_{m},a_{n_{1}}^{l}\right)$	$g_{m}$

**Table 2:** The decision utility information system.

R	$e_{1}$			…	$e_{l}$		
	$a_{1}^{1}$	…	$a_{n_{1}}^{1}$	…	$a_{1}^{l}$	…	$a_{n_{1}}^{l}$
$x_{1}$	$U^{1}\left(x_{1},a_{1}^{1}\right)$	…	$U^{1}\left(x_{1},a_{n_{1}}^{1}\right)$	…	$U\left(x_{1},a_{1}^{l}\right)$	…	$U^{l}\left(x_{1},a_{n_{1}}^{l}\right)$
$x_{2}$	$U^{1}\left(x_{2},a_{1}^{1}\right)$	…	$U^{1}\left(x_{2},a_{n_{1}}^{1}\right)$	…	$U^{l}\left(x_{2},a_{1}^{l}\right)$	…	$U^{l}\left(x_{2},a_{n_{1}}^{l}\right)$
…	…	…	…	…	…	…	…
$x_{m}$	$U^{1}\left(x_{m},a_{1}^{1}\right)$	…	$U^{1}\left(x_{m},a_{n_{1}}^{1}\right)$	…	$U^{l}\left(x_{m},a_{1}^{l}\right)$	…	$U^{l}\left(x_{m},a_{n_{1}}^{l}\right)$

**Table 3:** The loss function.

	$D_{A}^{k}$	$D_{H}^{k}$	$D_{R}^{k}$
$b_{A}$	$l_{AD_{A}^{k}}^{i}$	$l_{AH_{A}^{k}}^{i}$	$l_{AR_{A}^{k}}^{i}$
$b_{H}$	$l_{HD_{A}^{k}}^{i}$	$l_{HH_{A}^{k}}^{i}$	$l_{HR_{A}^{k}}^{i}$
$b_{R}$	$l_{RD_{A}^{k}}^{i}$	$l_{RH_{A}^{k}}^{i}$	$l_{RR_{A}^{k}}^{i}$

**Table 4:** FMEA-based decision information system.

R	$e_{1}$			…	$e_{l}$			$g_{k}$
	S	O	D	…	$a_{1}^{l}$	…	$a_{n_{1}}^{l}$	
$x_{1}$	$f^{1}\left(x_{1},a_{1}^{1}\right)$	…	$f^{1}\left(x_{1},a_{n_{1}}^{1}\right)$	…	$f^{l}\left(x_{1},a_{1}^{1}\right)$	…	$f^{l}\left(x_{1},a_{n_{1}}^{l}\right)$	$g_{1}$
$x_{2}$	$f^{1}\left(x_{2},a_{1}^{1}\right)$	…	$f^{1}\left(x_{2},a_{n_{1}}^{1}\right)$	…	$f^{l}\left(x_{2},a_{1}^{1}\right)$	…	$f^{l}\left(x_{2},a_{n_{1}}^{l}\right)$	$g_{2}$
…	…	…	…	…	…	…	…	…
$x_{m}$	$f^{1}\left(x_{m},a_{1}^{1}\right)$	…	$f^{1}\left(x_{m},a_{n_{1}}^{1}\right)$	…	$f^{l}\left(x_{m},a_{1}^{1}\right)$	…	$f^{l}\left(x_{m},a_{n_{1}}^{l}\right)$	$g_{m}$

**Table 5:** The utility matrix.

R	$e_{1}$			…	$e_{l}$		
	S	O	D	…	$a_{1}^{l}$	…	$a_{n_{1}}^{l}$
$x_{1}$	$v^{1}\left(x_{1},a_{1}^{1}\right)$	…	$v^{l}\left(x_{1},a_{n_{1}}^{1}\right)$	…	$v^{l}\left(x_{1},a_{1}^{1}\right)$	…	$v^{l}\left(x_{1},a_{n_{1}}^{l}\right)$
$x_{2}$	$v^{1}\left(x_{2},a_{1}^{1}\right)$	…	$v^{l}\left(x_{2},a_{n_{1}}^{1}\right)$	…	$v^{l}\left(x_{2},a_{1}^{1}\right)$	…	$v^{l}\left(x_{2},a_{n_{1}}^{l}\right)$
…	…	…	…	…	…	…	…
$x_{m}$	$v^{1}\left(x_{m},a_{1}^{1}\right)$	…	$v^{l}\left(x_{m},a_{n_{1}}^{1}\right)$	…	$v^{l}\left(x_{m},a_{1}^{1}\right)$	…	$v^{l}\left(x_{m},a_{n_{1}}^{l}\right)$

**Table 6:** The utility function.

	$D_{A}^{k}$	$D_{H}^{k}$	$D_{R}^{k}$
$b_{A}$	$l_{AD_{A}^{k}}^{i}$	$l_{AH_{A}^{k}}^{i}$	$l_{AR_{A}^{k}}^{i}$
$b_{H}$	$l_{HD_{A}^{k}}^{i}$	$l_{HH_{A}^{k}}^{i}$	$l_{HR_{A}^{k}}^{i}$
$b_{R}$	$l_{RD_{A}^{k}}^{i}$	$l_{RH_{A}^{k}}^{i}$	$l_{RR_{A}^{k}}^{i}$

**Table 7:** Incomplete information system of in-service aircraft structures.

Structure Name	No.	Design Granularity		Simulation Granularity		Event Granularity	
		Severity	Occurrence	Static Force	Fatigue Life	Frequency	Influence
Spoiler	A_1_	0.63	0.32	0	0	0.10	3
Aileron	A_2_	0.7	0.15	0.90	0.14	0.1	3
Fuselage	A_3_	0.7	0.15	0	0	0.17	5
Rudder	A_4_	0.5	0.15	0	0	0.17	5
Vertical Stabilizer	A_5_	0.7	0.15	0	0	0	0
Horizontal Stabilizer	A_6_	0.5	0.15	16.16	0.74	0.03	1
Elevator	A_7_	0.5	0.15	0	0	0.2	6
Engine	A_8_	0.9	0.4	0	0	0	0
Wing	A_9_	0.9	0.65	0	0	0	0
Landing Gear	A_10_	0.9	0.4	18.93	2.59	0.23	16.34
Flap	A_11_	0.5	0.15	30.31	4.575	0	0

**Table 8:** Preliminary results of the key factors from three different scenarios.

Structure Name	No.	Design Granularity	Simulation Granularity	Event Granularity
Spoiler	A_1_	1	1	1
Aileron	A_2_	1	−1	−1
Fuselage	A_3_	1	−1	−1
Rudder	A_4_	0	−1	0
Vertical Stabilizer	A_5_	−1	1	−1
Horizontal Stabilizer	A_6_	−1	−1	0
Elevator	A_7_	-1	−1	0
Engine	A_8_	−1	−1	0
Wing	A_9_	−1	−1	0
Landing Gear	A_10_	−1	0	−1
Flap	A_11_	−1	−1	−1

**Table 9:** The comparison results among the various algorithms.

	Loss Function	Classification	Risk Appetite Consideration	Consensus Consideration	Availability When Data Amount > 10
K_means	√	√	×	×	√
DBSCAN	√	√	×	×	√
HC	√	√	×	×	√
TOPSIS	×	×	×	√	√
Our method	√	√	√	√	√

## Data Availability

The original contributions presented in the study are included in the article; further inquiries can be directed to the corresponding authors.
